# Essential team science skills for biostatisticians on collaborative research teams

**DOI:** 10.1017/cts.2023.676

**Published:** 2023-11-06

**Authors:** Emily Slade, Ann M. Brearley, Adrian Coles, Matthew J. Hayat, Pandurang M. Kulkarni, Amy S. Nowacki, Robert A. Oster, Michael A. Posner, Gregory Samsa, Heidi Spratt, Jesse Troy, Gina-Maria Pomann

**Affiliations:** 1 Department of Biostatistics, University of Kentucky, Lexington, KY, USA; 2 Division of Biostatistics, School of Public Health, University of Minnesota, Minneapolis, MN, USA; 3 Global Biometrics and Data Sciences, Bristol Myers Squibb, Lawrence Township, NJ, USA; 4 Department of Population Health Sciences, School of Public Health, Georgia State University, Atlanta, GA, USA; 5 Global Data Sciences & Scientific Communications, Eli Lilly & Co., Indianapolis, IN, USA; 6 Department of Quantitative Health Sciences, Lerner Research Institute, Cleveland Clinic, Cleveland, OH, USA; 7 Division of Preventive Medicine, Department of Medicine, University of Alabama at Birmingham, Birmingham, AL, USA; 8 Department of Mathematics and Statistics, Villanova University, Villanova, PA, USA; 9 Department of Biostatistics and Bioinformatics, Duke University, Durham, NC, USA; 10 Department of Biostatistics and Data Science, School of Public and Population Health, University of Texas Medical Branch, Galveston, TX, USA

**Keywords:** Team science, collaboration, biostatistics, data science, clinical and translational research, training

## Abstract

**Introduction:**

Despite the critical role that quantitative scientists play in biomedical research, graduate programs in quantitative fields often focus on technical and methodological skills, not on collaborative and leadership skills. In this study, we evaluate the importance of team science skills among collaborative biostatisticians for the purpose of identifying training opportunities to build a skilled workforce of quantitative team scientists.

**Methods:**

Our workgroup described 16 essential skills for collaborative biostatisticians. Collaborative biostatisticians were surveyed to assess the relative importance of these skills in their current work. The importance of each skill is summarized overall and compared across career stages, highest degrees earned, and job sectors.

**Results:**

Survey respondents were 343 collaborative biostatisticians spanning career stages (early: 24.2%, mid: 33.8%, late: 42.0%) and job sectors (academia: 69.4%, industry: 22.2%, government: 4.4%, self-employed: 4.1%). All 16 skills were rated as at least somewhat important by > 89.0% of respondents. Significant heterogeneity in importance by career stage and by highest degree earned was identified for several skills. Two skills (“regulatory requirements” and “databases, data sources, and data collection tools”) were more likely to be rated as absolutely essential by those working in industry (36.5%, 65.8%, respectively) than by those in academia (19.6%, 51.3%, respectively). Three additional skills were identified as important by survey respondents, for a total of 19 collaborative skills.

**Conclusions:**

We identified 19 team science skills that are important to the work of collaborative biostatisticians, laying the groundwork for enhancing graduate programs and establishing effective on-the-job training initiatives to meet workforce needs.

## Introduction

In clinical and translational research, biostatisticians, bioinformaticians, epidemiologists, data scientists, and other quantitative experts are integral parts of multidisciplinary teams conducting data-driven research. Their contributions span from study conception to dissemination, with responsibilities such as refining study aims, contributing to study design, analyzing data, interpreting results, and sometimes implementation. These quantitative experts work to ensure research findings are valid, reliable, impactful, and generalizable to the broader population. There are many different specialized areas of quantitative expertise needed to conduct data-driven research [1]. While graduate programs in quantitative science fields are often focused primarily on training students to gain appropriate technical and methodological expertise, there is a recognized need within the research community to develop methods to train quantitative experts to thrive within multidisciplinary teams [2,3]. The purpose of this paper is to present survey results that build upon previous research in order to help the community develop appropriate training for this workforce. While the focus of this work is on collaborative biostatisticians, we posit that the team science skills and training methods discussed are highly transferrable to other quantitative experts such as data scientists, statisticians, bioinformaticians, epidemiologists, engineers, implementation scientists, and others.

Current literature has established that biostatisticians facilitate innovation in translational science by working in interdisciplinary teams to develop new solutions and leverage statistical knowledge to identify novel approaches to diagnosis, treatment, and prevention of disease [4,5]. Because they often have experience working with a wide range of data types, designs, and research questions, biostatisticians are often required to facilitate communication with teams comprising researchers in varied fields [6]. In order to contribute meaningfully to an interdisciplinary research team, a biostatistician must not only be proficient in statistical theory, methods, and data analysis, but they must also be able to work collaboratively, effectively communicate with researchers from diverse fields and backgrounds, and effectively interpret results for various audiences [4,7–9]. Effective communication and interpretation for various audiences requires the biostatistician to develop subject matter knowledge for use in scientific reasoning. Typical graduate training for biostatisticians focuses on statistical theory and data analysis with limited training in communication, collaboration, and leadership [10], despite the fact that two of the seven recommendations by the American Statistical Association (ASA) for master’s programs comprise communication, collaboration, and leadership [11]. Others have also addressed how ethics should be incorporated more into traditional graduate training in statistics [12]. In order to fill these gaps with on-the-job training programs or revise graduate curricula to more comprehensively cover collaborative skills, it is necessary to first identify the specific collaborative skills that are important to the work of biostatisticians on collaborative research teams.

Recent work by Pomann et al. proposed 16 competencies considered to be essential to the work of collaborative biostatisticians [13]. This list of competencies for this workforce was grouped into three broad categories: (1) communication and leadership, (2) clinical and domain knowledge, and (3) statistical expertise [13]. The focus of these competencies was not on technical and methodological skills, but instead on the overarching team science competencies related to these categories. For example, the statistical expertise category includes competencies such as coding, reproducibility, and statistical analysis plans, but it does not include specific statistical methodologies.

The competencies and skills proposed by Pomann et al. were curated in collaboration with the leadership of biostatistics units at 13 different institutions and companies but have not yet been evaluated to assess if these skills are essential to the work of collaborative biostatisticians across job sectors and to quantify the relative importance of each skill [13]. Recent work by Satagopan & Mazumdar proposed similar core competencies fundamental to team science success of collaborative biostatisticians: (1) active listening, (2) communication, and (3) networking, but again, formal evaluation of these competencies has not yet been completed [2]. In this paper, we build on previous work to define team science skills and provide the first formal evaluation of the importance of these skills in the collaborative biostatistics workforce, both overall and across career stages, highest degrees earned, and job sectors.

## Methods

### Development of definitions

In order to effectively survey current collaborative biostatisticians regarding the use of these skills in their work, we built upon recent literature and observations from our own practice to define specific skills that can be evaluated. The development of skill definitions and refinement of phrasing for this study was conducted by a workgroup of collaborative biostatisticians, who are professionals with current roles in at least one of 10 academic institutions and companies including seven universities, one large research hospital, and two pharmaceutical companies. Some workgroup members have previous professional experience in academic and private-sector settings. Sixteen skills were defined, adapted from Pomann *et al*. [13], and concrete descriptions were created for all 16 skills (Table 1). The skill descriptions were developed through focused deliberation among workgroup members with the goal of creating definitions that were clear but still short enough to be read and processed quickly while completing a survey.

**Table 1.** Team science skills considered to be important to the work of collaborative biostatisticians

Skill definitions and descriptions as used in the collaborative biostatistician survey	
*Skill*	*Description*
Databases, data sources, and data collection tools	Understand nuances of working with databases, data sources, and data collection tools, including their advantages/ limitations for answering clinical/scientific questions
Developing clinical/scientific domain knowledge	Learn about a biomedical domain as needed for a given project to critically assess and interpret scientific results
Regulatory requirements	Understand, implement, and explain relevant data security and regulatory requirements
Institutional structure	Navigate one’s institutional infrastructure, processes, and funding mechanisms
Statistical analysis plans	Write and critique statistical analysis plans with sufficient detail to ensure replicable fulfillment of study objectives
Reproducibility	Utilize a documentation process for project reproducibility including data storage, coding, analysis plans, reports, etc. so that the project can be reproduced by others
Coding	Write code to manage data and/or implement statistical methods using accurate and efficient coding practices
Literature review	Conduct review of literature and background information to identify gaps in scientific knowledge to motivate a given project
Learning new statistical methods	Learn and implement unfamiliar statistical methods needed for a given project
Professional correspondence	Correspond with collaborators promptly, appropriately, and clearly
Time/project management	Complete projects while planning for and managing multiple projects simultaneously
Effective meeting strategy	Facilitate effective discussions with clinical/scientific collaborators to achieve meeting goals and develop follow-up steps
Scientific communication	Effectively communicate scientific/statistical concepts with collaborators
Presenting results	Present results formally, both verbally and in writing, in a clear and non-technical manner
Using strong statistical voice	Advocate and negotiate for good and ethical statistical practices including integrating and resolving differing scientific approaches
Collaboration with analytic colleagues	Navigate a network of colleagues to identify additional methodological expertise and solicit guidance or collaboration as needed
**Additional skills identified by respondents on the collaborative biostatistician survey**	
*Skill*	*Description*
Diversity, equity, inclusion, and accessibility	Incorporate diverse perspectives and ensure fair representation both in the research process and in the workplace
Professional development	Actively shape one’s career trajectory, including continuing education, setting boundaries, and avoiding burnout
Mentoring and supervision	Serve as a formal mentor to trainees, a supervisor to employees, or a peer mentor to colleagues with diverse training backgrounds

The top section contains 16 skills used in the collaborative biostatistician survey. Skills were adapted from Pomann *et al*. [13], and descriptions of these skills were created for this study. The bottom section contains 3 skills that were not included in the collaborative biostatistician survey but were also identified as important in the collaborative biostatistics workforce.

In order to survey current collaborative biostatisticians about the relative importance of these skills, we also worked to formulate a precise definition of the term “collaborative biostatistician.” We developed the following working definition for use in the survey: “A “collaborative biostatistician” is defined as a quantitative expert for whom one of their primary roles is collaborating with non-statisticians on projects that are intended to answer a biomedical question rather than to develop new statistical methodology. We consider a “biomedical question” to be a wide net that applies to any industry in which biostatisticians work, but we are specifically trying to capture those whose primary focus is on applied research rather than methods research.”

### Survey development

The Research Electronic Data Capture (REDCap) platform was used to develop and administer the survey [14], and this study was approved by the Institutional Review Board at the University of Kentucky (#74933). After initial development of the collaborative biostatistician survey, eight individuals from outside the survey development workgroup piloted the survey to provide feedback on the clarity of skill definitions and survey questions via free response. The individuals who piloted the survey were selected to provide perspectives from a range of job sectors in which collaborative biostatisticians work including academia [*n* = 1], industry (both for-profit [*n* = 1] and non-profit [*n* = 2]), government [*n* = 3], and self-employment [*n* = 1]. The wording of skill definitions and survey questions were then revised for clarity by the survey development workgroup in accordance with the pilot feedback.

Respondents to the collaborative biostatistician survey were first asked to provide information about their demographics, their graduate training, and their current job sector (academia/academic medical center, government, industry (for-profit), industry (non-profit), and self-employment). Participants were then presented with the 16 revised skill descriptions (adapted from Pomann *et al*. [13]) and accompanying definitions (Table 1) and were asked to “Rate the importance of each of the following 16 skills in your current work as a collaborative biostatistician” on a 4-point scale (not important, somewhat important, important, absolutely essential). Participants were then presented with a free response text box and asked, “If there are any skills that you consider to be important/essential to your current work as a collaborative biostatistician that were not included on the list above, please list them here.”

### Study population and survey distribution

Inclusion criteria for the collaborative biostatistician survey were currently working as collaborative biostatistician in any job sector and having completed a graduate degree. The survey was distributed through several advertising channels including discussion boards for American Statistical Association sections; flyers at the International Biometric Society Eastern North American Region conference in Nashville, TN; Twitter; LinkedIn; collaborative biostatistician networks such as the Biostatistics, Epidemiology, and Research Design Special Interest Group (BERD SIG); and emails to leaders of collaborative biostatistics groups. The survey was open from March 17 to May 5, 2023.

### Analysis of survey data

All analyses were performed using R version 4.2.0 [15]. Available case analysis was used for all analyses, with the amount of missing data reported. Counts and proportions were used to summarize participants’ ratings of each of the 16 skills as not important, somewhat important, important, or absolutely essential to their current work as a collaborative biostatistician. For analysis purposes, skills with at least 75% of participants rating the skill as somewhat important, important, or absolutely essential were considered to be confirmed as important to the work of collaborative biostatisticians.

Next, we assessed heterogeneity in the importance of each skill by career stage. Individual participants identified their own career stage as early, mid, or late career, representing 0–5, 6–15, or 16+ years spent working as a collaborative biostatistician, respectively. We selected these categories to represent the expected difference in roles performed within each career stage: within the first five years, one often spends time building their skills; within the next ten years, one often plays a lead role in implementing these skills; and beyond 15 years, one often takes on more senior leadership roles. Descriptive results are displayed for the proportion of participants rating each skill as absolutely essential, stratified by career stage. For each skill, a chi-squared test was used to quantify the association between career stage and rating the skill as absolutely essential (yes/no) in one’s current work as a collaborative biostatistician. Skills with *p* < 0.05 were considered to differ significantly in importance by career stage, with no adjustment made for multiple testing. The same methodology was used to assess heterogeneity in the importance of skills by the highest degree earned.

The same methodology was also used to assess heterogeneity in the importance of skills by job sector, with a focus on comparing the academia/academic medical center sector to the industry sector (combining for-profit and non-profit industries). Descriptive results are reported for all job sectors (academia/academic medical center, industry, government, and self-employed), but due to the small number of respondents who work in government or are self-employed, hypothesis testing for heterogeneity of importance by job sector was restricted to comparing the perceived importance of each skill between collaborative biostatisticians working in academia and industry.

Responses to the open-ended question prompting participants to identify any additional skills that they consider to be important/essential in their current work as a collaborative biostatistician were reviewed and deemed eligible for reporting if they met the following criteria: (1) suggested skill does not overlap with existing list of skills; (2) suggested skill is not a statistical methodology; and (3) suggested skill is plausible to have importance in the work of collaborative biostatisticians, as considered by the survey workgroup. Suggested skills meeting these criteria were then reviewed for overlap with each other and, when possible, were consolidated into themes with overarching skill definitions.

## Results

Of the 371 people who met inclusion criteria and consented to be included in the study, 344 completed at least one question about the importance of skills on the collaborative biostatistician survey. One participant’s response was excluded due to a suspected data entry error in the years spent as a collaborative biostatistician, resulting in 343 people in the analytic dataset from which all results were calculated. Survey participants spanned career stages (0–5 years: 24.2%, 6–15 years: 33.8%, 16 + years: 42.0%) and highest earned degrees (doctorate: 59.5%, master’s: 40.5%) (Table 2). The majority of participants were currently working in academia (69.4%) or industry (12.0% for-profit, 10.2% non-profit), with 4.4% working in government and 4.1% self-employed (Table 2). Full demographic information can be found in Table 2.

**Table 2.** Characteristics of survey participants (*n* = 343)

Variable	N (%)
Age	
18-24	6 (1.8%)
25-34	74 (21.9%)
35-44	85 (25.1%)
45-54	88 (26.0%)
55-64	54 (16.0%)
65+	31 (9.2%)
Gender identity	
Woman	169 (50.3%)
Man	162 (48.2%)
Transgender woman	0 (0.0%)
Transgender man	1 (0.3%)
Non-binary/Gender fluid	4 (1.2%)
Race	
American Indian or Alaskan Native	0 (0.0%)
Asian	40 (12.3%)
Black or African American	9 (2.8%)
Native Hawaiian or other Pacific Islander	0 (0.0%)
White	270 (82.8%)
Two or more races	7 (2.1%)
Ethnicity	
Not of Hispanic or Latino/a origin	313 (95.4%)
Hispanic, Latino/a origin	15 (4.6%)
Highest degree	
Doctorate	204 (59.5%)
Master’s	139 (40.5%)
Graduate certificate	0 (0.0%)
Years spent as a collaborative biostatistician	
0-5	83 (24.2%)
6-15	116 (33.8%)
16+	144 (42.0%)
Job sector	
Academia/academic medical center	238 (69.4%)
Industry (for-profit)	41 (12.0%)
Industry (non-profit)	35 (10.2%)
Government	15 (4.4%)
Self-employed	14 (4.1%)

The following variables had missing data: age *(n* = 5), gender identity (*n* = 7), race (*n* = 17), ethnicity (*n* = 15). Denominators for percentages include only non-missing responses.

Three skills – “using strong statistical voice,” “scientific communication,” and “presenting results” – were rated as somewhat important, important, or absolutely essential by 100% of participants (Table 3). All remaining skills were rated as at least somewhat important by > 97.5% of participants except regulatory requirements (92.2%), institutional structure (90.3%), and literature review (89.4%) (Table 3). For descriptions of each of these skills, see Table 1.

**Table 3.** Importance of each skill in all participants’ current work as a collaborative biostatistician, *n* = 343

Skill	Not important	Somewhat important	Important	Absolutely essential
Databases, data sources, and data collection tools	8 (2.4%)	37 (11.2%)	103 (31.1%)	183 (55.3%)
Developing clinical/ scientific domain knowledge	4 (1.2%)	55 (16.6%)	149 (44.9%)	124 (37.3%)
Regulatory requirements	26 (7.8%)	102 (30.7%)	125 (37.7%)	79 (23.8%)
Institutional structure	32 (9.7%)	126 (38.1%)	129 (39.0%)	44 (13.3%)
Statistical analysis plans	4 (1.2%)	16 (4.8%)	72 (21.7%)	240 (72.3%)
Reproducibility	2 (0.6%)	20 (6.0%)	114 (34.3%)	196 (59.0%)
Coding	2 (0.6%)	11 (3.3%)	61 (18.4%)	257 (77.6%)
Literature review	35 (10.6%)	102 (30.8%)	133 (40.2%)	61 (18.4%)
Learning new statistical methods	3 (0.9%)	37 (11.2%)	128 (38.7%)	163 (49.2%)
Professional correspondence	2 (0.6%)	11 (3.3%)	98 (29.5%)	221 (66.6%)
Time/project management	1 (0.3%)	14 (4.2%)	78 (23.6%)	238 (71.9%)
Effective meeting strategy	2 (0.6%)	40 (12.1%)	139 (42.0%)	150 (45.3%)
Scientific communication	0 (0.0%)	4 (1.2%)	77 (23.3%)	250 (75.5%)
Presenting results	0 (0.0%)	4 (1.2%)	66 (20.2%)	257 (78.6%)
Using strong statistical voice	0 (0.0%)	26 (7.9%)	112 (33.8%)	193 (58.3%)
Collaboration with analytic colleagues	3 (0.9%)	45 (13.6%)	156 (47.0%)	128 (38.6%)

Denominators for percentages include only the participants who responded with an importance rating for the given skill. All skills had missing data ranging from *n* = 11 to *n* = 16 participants.

**Figure 1.**
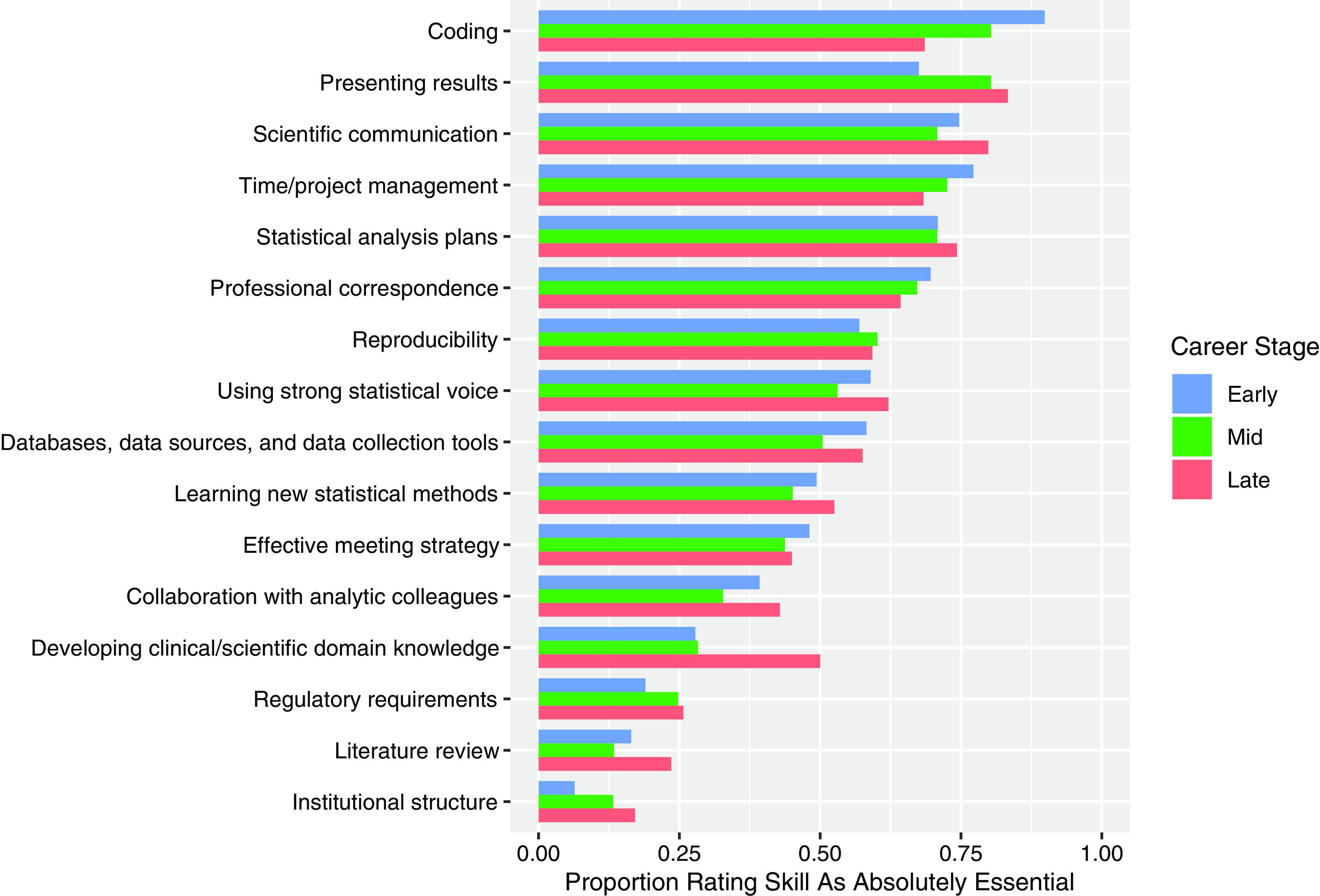
Proportion of respondents rating each skill as absolutely essential by career stage. Blue represents early-career collaborative biostatisticians (0–5 years, *n* = 83), green represents mid-career collaborative biostatisticians (6–15 years, *n* = 116), and pink represents late-career collaborative biostatisticians (16+ years, *n* = 144). Skills are listed in descending order based on the overall proportion rating the skill as absolutely essential across career stages.

Early-career respondents (working as a collaborative biostatistician for 5 years or less) rated “coding,” “time/project management,” and “scientific communication” as the most important skills in their current work (89.9%, 77.2%, and 74.7%, respectively, rated as absolutely essential) (Fig. 1). Mid-career respondents (working as a collaborative biostatistician for 6-15 years) rated “presenting results,” “coding,” and “time/project management” as the most important skills in their current work (80.4%, 80.4%, and 72.6%, respectively, rated as absolutely essential) (Fig. 1). Late-career respondents (working as a collaborative biostatistician for 16 years or longer) rated “presenting results,” “scientific communication,” and “statistical analysis plans” as the most important skills in their current work (83.3%, 79.9%, and 74.3%, respectively, rated as absolutely essential) (Fig. 1). For the importance rating of all skills by career stage, see Fig. 1.

The skill with the most significant heterogeneity in importance by career stage was “developing clinical/scientific domain knowledge” *(p* < 0.001, Supplemental Table 1), rated as absolutely essential by 50.0% of late-career respondents but only 27.8% and 28.3% of early and mid-career respondents, respectively (Fig. 1, Supplemental Table 1). “Coding” also had a notably high degree of heterogeneity (*p* = 0.001, Supplemental Table 1), with the perceived importance waning with increased duration in the field (89.9% of early career, 80.4% of mid-career, and 68.6% of late-career respondents rating “coding” as absolutely essential) (Fig. 1, Supplemental Table 1). Finally, the importance of “presenting results” also had significant heterogeneity across career stages (*p* = 0.022, Supplemental Table 1), exhibiting the opposite pattern: perceived importance was higher for participants of more advanced career stages (67.5% of early career, 80.4% of mid-career, and 83.3% of late-career respondents rating “presenting results” as absolutely essential) (Fig. 1, Supplemental Table 1).

**Figure 2.**
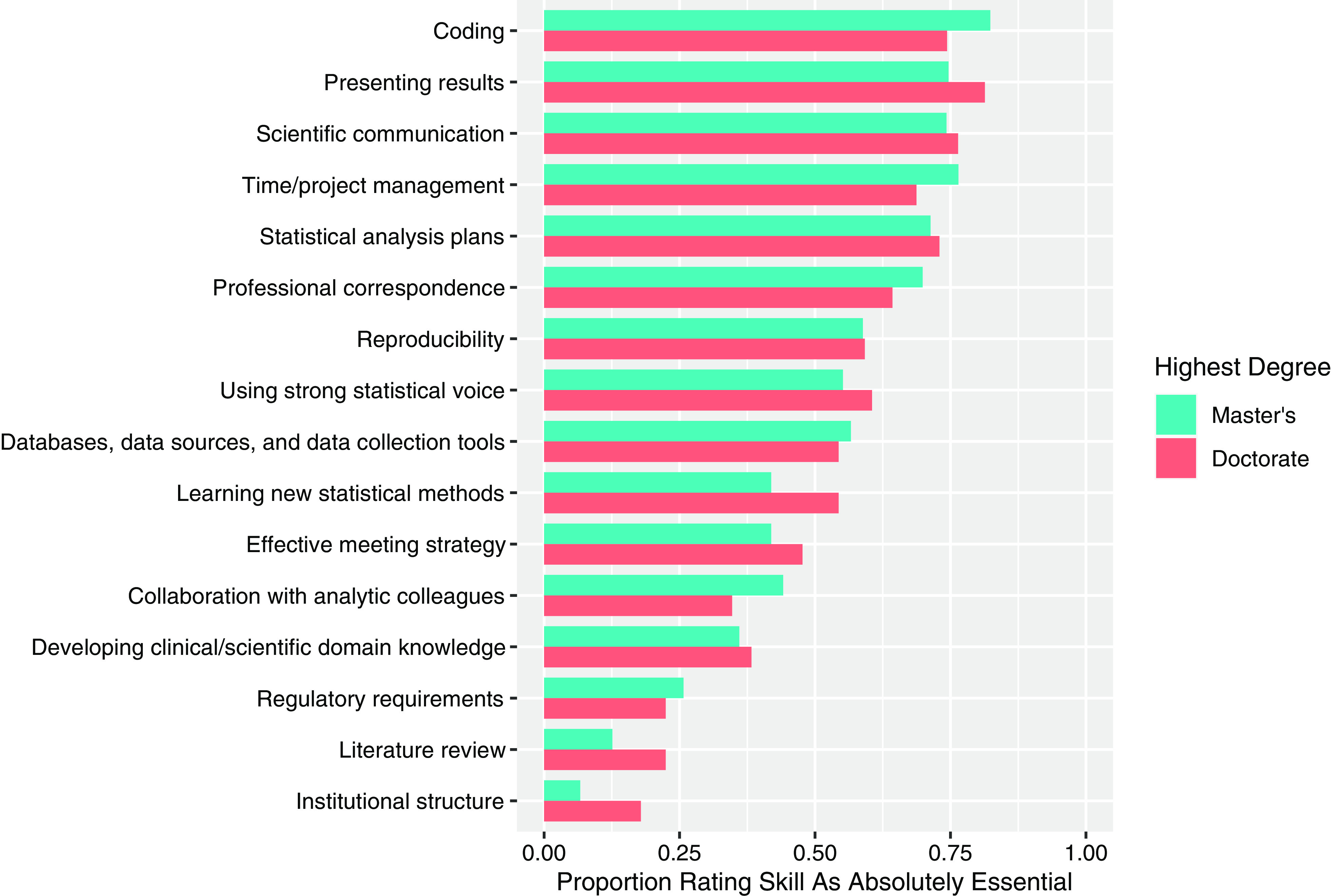
Proportion of respondents rating each skill as absolutely essential by highest degree earned. Turquoise represents master’s degree (*n* = 139), and pink represents doctoral degree (*n* = 204). Skills are listed in descending order based on the overall proportion rating the skill as absolutely essential across degrees earned.

Three skills (“institutional structure,” “literature review,” and “learning new statistical methods”) were rated as absolutely essential by a greater proportion of respondents with a doctoral degree than by those whose highest degree was a master’s degree (*p* = 0.005, *p* = 0.033, *p* = 0.034, respectively) (Supplemental Table 2). Of those with a doctoral degree, 54.4% of respondents rated “learning new statistical methods” as absolutely essential, as compared to 41.9% of respondents whose highest degree was a master’s degree (Fig. 2). While the proportion of respondents rating “institutional structure” and “literature review” as absolutely essential was higher amongst those with a doctoral degree (17.9% and 22.4%, respectively) than among those whose highest degree is a master’s degree (6.7% and 12.6%, respectively), the perceived importance of these skills was relatively low for both groups (Fig. 2). For the importance rating of all skills by highest degree earned, see Fig. 2.

**Figure 3.**
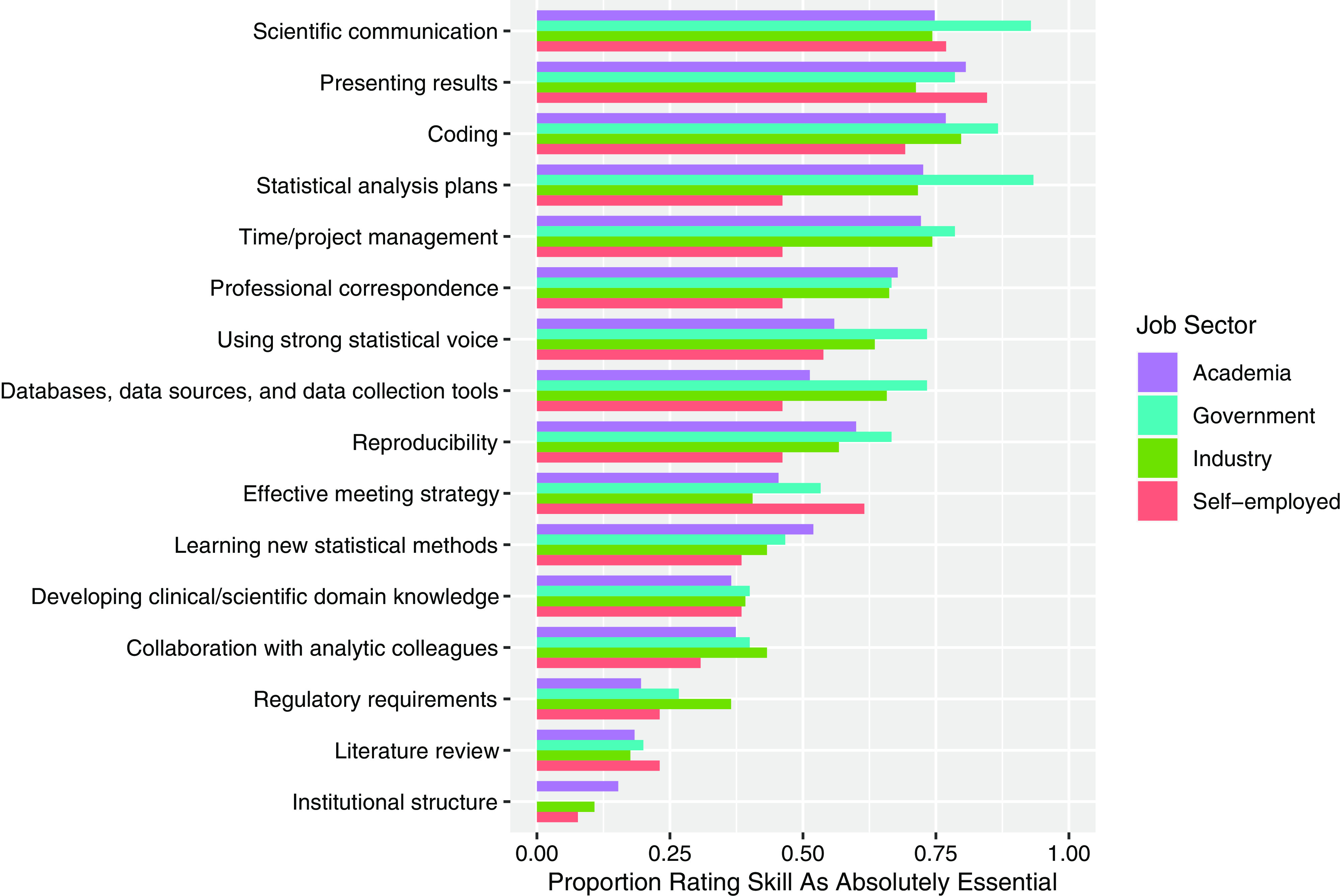
Proportion of respondents rating each skill as absolutely essential by job sector. Purple represents academia/academic medical centers (*n* = 238), blue represents government (*n* = 15), green represents for-profit and non-profit industry (*n* = 76), and pink represents self-employed (*n* = 14). Skills are listed in descending order based on the overall proportion rating the skill as absolutely essential across job sectors.

Participants working in academia or at an academic medical center rated “presenting results,” “coding,” and “scientific communication” as the most important skills in their current work (80.6%, 76.9%, and 74.8%, respectively, rated as absolutely essential) (Fig. 3). Those working in government rated “statistical analysis plans,” “scientific communication,” and “coding” as the most important (93.3%, 92.9%, and 86.7%, respectively, rated as absolutely essential) (Fig. 3). Those working in industry rated “coding,” “time/project management,” and “scientific communication” as the most important (79.7%, 74.3%, and 74.3%, respectively, rated as absolutely essential) (Fig. 3). Finally, those who were self-employed rated “presenting results,” “scientific communication,” and “coding” as the most important (84.6%, 76.9%, and 69.2%, respectively, rated as absolutely essential) (Fig. 3). For the importance rating of all skills by job sector, see Fig. 3.

For two skills, the proportion rating the skill as absolutely essential was significantly different between those working in academia and those working in industry. The “regulatory requirements” skill, defined as “understand, implement, and explain relevant data security and regulatory requirements,” was less likely to be rated as absolutely essential by those in academia (19.6%) than by those in industry (36.5%) (*p* = 0.005, Supplemental Table 3). Similarly, the “databases, data sources, and data collection tools” skill was also less likely to be rated as absolutely essential by those in academia (51.3%) than by those in industry (65.8%) (*p* = 0.043, Supplemental Table 3). This skill is defined as “understanding nuances of working with databases, data sources, and data collection tools, including their advantages/ limitations for answering clinical/scientific questions” (Table 1).

Sixty-three participants (18.4%) made suggestions for skills that they considered to be important to the work of collaborative biostatisticians but not included in the survey. Of these 63 participants, 65.1% had a doctorate degree, and 34.9% had a master’s degree as their highest degree. Those who suggested additional skills tended to be more advanced in their career (17.5% early career, 28.6% mid-career, 54.0% late career) than the overall survey respondents. Of the suggestions for additional skills, three themes emerged for skills that met inclusion criteria for reporting, including (1) diversity, equity, inclusion, and accessibility (DEIA); (2) professional development; and (3) mentoring and supervision. Similar to the first suggested skill, Satagopan & Mazumdar also listed “embracing diversity” as key to the team science of biostatistics collaborations [2]. Based on participant suggestions and our own assessment, the DEIA skill encompasses two main dimensions. Firstly, it encompasses aspects related to data collection and analysis, where individuals with DEIA proficiency are adept at preventing, recognizing, and addressing biases in data; ensuring fair representation; and incorporating diverse perspectives into the research process. Secondly, the DEIA skill extends to aspects related to the workplace, where individuals with this skill foster inclusive and welcoming environments, advocate for equitable practices, and promote accessibility for all members of the team. The second new skill suggestion, professional development, includes actively shaping one’s career trajectory, such as engaging in continuing education, mastering the art of setting boundaries, avoiding burnout, and “managing up,” which is defined as understanding how to provide value to one’s company/institution [16]. The third new skill suggestion is mentoring and supervision, recognizing that although mentoring mentees and supervising employees involve distinct responsibilities, they are interconnected enough to be classified within the same overarching skill category. As a collaborative biostatistician gains experience, they will often need to train and mentor others. Additionally, the team science nature of the work requires them to be able to serve as a formal mentor or peer mentor for colleagues with diverse training backgrounds. Therefore, formal training in these skills does seem imperative for this workforce. Due to these findings, we propose an updated list of skills to now include 19, and the three additional skill definitions and descriptions are provided in Table 1.

## Discussion

This study builds upon past work by Pomann et al. that proposed 16 non-methodological skills that are considered to be important to the work of collaborative biostatisticians [13]. This is the first study to empirically assess the importance of these skills overall and by career stage, highest degree earned, and job sector, and to survey current collaborative biostatisticians to identify any non-methodological skills that may be missing from this list. For all 16 skills, the vast majority of survey respondents (>89%) rated the skill as somewhat important, important, or absolutely essential in their current work, confirming that the skills proposed by Pomann et al. [13] are, in fact, important to the work of collaborative biostatisticians who responded to this survey. Our survey results also identified three additional skills that we consider to be important to the work of collaborative biostatisticians: DEIA, professional development, and mentoring and supervision.

This study has important implications for several audiences. First, educators and directors of graduate programs in statistics and biostatistics should use the data from this study to inform graduate curricula (both classroom-based learning and experiential opportunities such as internships) to appropriately equip students with the skills needed to succeed as a collaborative biostatistician. The relative importance of skills across job sectors also provides valuable information for trainees exploring career options in different sectors. For example, the “regulatory requirements” skill, defined as “understand, implement, and explain relevant data security and regulatory requirements,” is rated as nearly twice as essential in industry than in academia. Mentors can also use this information to guide students toward training opportunities that are aligned with the type of career that they wish to pursue.

This study also provides important information for early-career collaborative biostatisticians and their supervisors or mentors who may wish to examine the relative importance of skills across career stages as they consider targeted professional development opportunities throughout their career or their mentees’ careers. Importantly, supervisors should ensure that collaborative biostatisticians have designated time to build these skills [17]. We expect that some differences in the importance of skills across career stages are attributable simply to differences in job requirements; for example, the importance of “coding” was highest among early-career collaborative biostatisticians and wanes throughout one’s career. However, we also expect that some differences in the importance of skills across career stages could be due to late-career respondents having more insight about how the discipline of collaborative biostatistics is most effectively practiced, insights that may not yet be recognized by those early in their careers. For example, we posit that “developing clinical/scientific domain knowledge” is critical to the effective practice of collaborative biostatistics at any career stage, but the results of this survey indicate that the importance of this skill is recognized more as one advances in their career. Future work will explore existing training opportunities to identify gaps where new areas for training would have the greatest opportunity for impact.

Several suggestions made by participants for additional important skills were considered by our workgroup to be tasks where successfully performing the task would require mastery of multiple skills listed in Table 1. For example, budgeting for statistical analyses was suggested by multiple participants as an important skill. We agree that budgeting is important for many collaborative biostatisticians, but we posit that proficiency in the existing skills of time/project management, institutional structure, and statistical analysis plans is sufficient to be successful in budgeting. This highlights an important note on the 16 skills presented in Table 1 and three additional skills identified in this study – these skills should not be trained in isolation or expected to be completed in isolation; rather, many tasks that collaborative biostatisticians perform require the integration of multiple skills. There are likely many other activities and skills that will be defined as we continue to develop the workforce of collaborative biostatisticians. We hypothesize that the 19 skills identified in this study can be considered as a foundation needed to succeed at fundamental activities required for collaborative biostatisticians, but more work is required to confirm this hypothesis.

Of note, many participants in this study suggested additional skills that we considered to be already covered by the original list of 16 skills used for this study. This indicates that one weakness of this study is that survey respondents may not have fully understood the skill definitions that were provided to them, and as such, the importance of these skills may be underestimated by our survey. For example, many additional skills suggested by survey respondents would fall under what we consider to be “using strong statistical voice,” such as conflict resolution, compromising with collaborators, assertiveness, and understanding that there are oftentimes multiple ways to analyze data to answer a research question. In response to this concern, future work will focus on defining the skills in Table 1 in more detail so that targeted trainings can be developed for each skill.

This study represents a convenience sample of collaborative biostatisticians, and less than one-third of survey respondents were currently working in job sectors outside of academia. Specific efforts were made to reach collaborative biostatisticians outside of academia (such as advertising the survey on the ASA Biopharmaceutical Section and ASA Government Statistics section discussion boards), but it is still likely that the professional networks and channels used for survey dissemination contain a disproportionate number of academic collaborative biostatisticians. In particular, there were a small number of survey responses from government (*n* = 15) and self-employed (*n* = 14) collaborative biostatisticians. Descriptive results are reported for these job sectors, but results should not be generalized (for government and self-employed collaborative biostatisticians) due to the small sample size in these groups. Additionally, master’s-level biostatisticians may be underrepresented in this survey due to more limited funding for professional society membership and conference attendance than doctoral-level biostatisticians. Further, while we believe that much of this work can be extended to other quantitative specialties such as data science, epidemiology, etc., future work is needed to understand if this is true and to identify which skills are most important in these related fields.

Collaborative biostatistics positions are different across and even within job sectors with respect to the proportion of one’s time that is spent on collaborative research as opposed to methodological research, administration/management, teaching, or other duties not considered to be part of working on applied, interdisciplinary research teams. We did not ask participants to report the proportion of their job that involves collaborative biostatistics work; rather, we asked them only to indicate whether one of their primary roles is what we consider to be collaborative biostatistics work (i.e., working with non-statisticians on projects that are intended to answer a biomedical question rather than to develop new statistical methodology). Therefore, we expect that our participant pool contains both individuals who work entirely as collaborative biostatisticians and those who spend some but not all of their time as such.

Survey respondents were predominantly White (82.8%) and not of Hispanic or Latino/a origin (95.4%). While it is possible that White and non-Hispanic or Latino/a collaborative biostatisticians were more likely to respond to our survey, it is also possible that these groups are overrepresented in the field of collaborative biostatistics. Thus, there could be a need for increased and improved pipeline programs to foster diversity in the collaborative biostatistics workforce. By actively fostering a more diverse talent pool, we can ensure that a broader range of perspectives and experiences are represented.

The demand for data-driven research and evidence-based policy-making has created high demand across sectors for biostatisticians who excel at collaborating and communicating with diverse teams of people [7,18]. With this rising demand, it becomes crucial to understand the essential characteristics of this work, laying the groundwork for enhancing graduate programs and establishing effective on-the-job training initiatives to meet workforce needs. By nurturing these skills and knowledge, collaborative biostatisticians can contribute even more effectively to clinical and translational science, thereby advancing patient outcomes and public health.

## Supporting information

Slade et al. supplementary material Slade et al. supplementary material

## Supplementary material

The supplementary material for this article can be found at https://doi.org/10.1017/cts.2023.676.
