# Supplementary material for: Essential team science skills for biostatisticians on collaborative research teams
Source: J Clin Transl Sci. 2023 Nov 6;7(1):e243. doi: 10.1017/cts.2023.676 (PMC10685263; doi:10.1017/cts.2023.676)
Supplement: Slade et al. supplementary material [file S2059866123006763sup001.docx]

**Supplemental Table 1**: Heterogeneity in rating each skill as absolutely essential by career stage

| **Skill** | **Early Career (n=83)** | **Mid-Career (n=116)** | **Late Career (n=144)** | **p-value** |
| --- | --- | --- | --- | --- |
| Databases, data sources, and data collection tools | 46 (58.2%) | 57 (50.4%) | 80 (57.6%) | 0.441 |
| Developing clinical/scientific domain knowledge | 22 (27.8%) | 32 (28.3%) | 70 (50.0%) | <0.001 |
| Regulatory requirements | 15 (19.0%) | 28 (24.8%) | 36 (25.7%) | 0.509 |
| Institutional structure | 5 (6.4%) | 15 (13.3%) | 24 (17.1%) | 0.082 |
| Statistical analysis plans | 56 (70.9%) | 80 (70.8%) | 104 (74.3%) | 0.786 |
| Reproducibility | 45 (57.0%) | 68 (60.2%) | 83 (59.3%) | 0.903 |
| Coding | 71 (89.9%) | 90 (80.4%) | 96 (68.6%) | 0.001 |
| Literature review | 13 (16.5%) | 15 (13.4%) | 33 (23.6%) | 0.102 |
| Learning new statistical methods | 39 (49.4%) | 51 (45.1%) | 73 (52.5%) | 0.506 |
| Professional correspondence | 55 (69.6%) | 76 (67.3%) | 90 (64.3%) | 0.711 |
| Time/project management | 61 (77.2%) | 82 (72.6%) | 95 (68.3%) | 0.368 |
| Effective meeting strategy | 38 (48.1%) | 49 (43.8%) | 63 (45.0%) | 0.834 |
| Scientific communication | 59 (74.7%) | 80 (70.8%) | 111 (79.9%) | 0.246 |
| Presenting results | 52 (67.5%) | 90 (80.4%) | 115 (83.3%) | 0.022 |
| Using strong statistical voice | 46 (59.0%) | 60 (53.1%) | 87 (62.1%) | 0.346 |
| Collaboration with analytic colleagues | 31 (39.2%) | 37 (32.7%) | 60 (42.9%) | 0.257 |

**Caption:** Career stages are defined as working as a collaborative biostatistician for 0-5 years (early), 6-15 years (mid), or 16+ years (late). Denominators for percentages include only the participants who responded with an importance rating for the given skill. P-value represents the association between career stage and rating the skill as “Absolutely essential” (yes/no) from a chi-squared test.

**Supplemental Table 2**: Heterogeneity in rating each skill as absolutely essential by highest degree earned

| **Skill** | **Master’s (n=139)** | **Doctorate (n=204)** | **p-value** |
| --- | --- | --- | --- |
| Databases, data sources, and data collection tools | 77 (56.6%) | 106 (54.4%) | 0.769 |
| Developing clinical/scientific domain knowledge | 49 (36.0%) | 75 (38.3%) | 0.765 |
| Regulatory requirements | 35 (25.7%) | 44 (22.4%) | 0.575 |
| Institutional structure | 9 (6.7%) | 35 (17.9%) | 0.005 |
| Statistical analysis plans | 97 (71.3%) | 143 (73.0%) | 0.839 |
| Reproducibility | 80 (58.8%) | 116 (59.2%) | >0.999 |
| Coding | 112 (82.4%) | 145 (74.4%) | 0.113 |
| Literature review | 17 (12.6%) | 44 (22.4%) | 0.033 |
| Learning new statistical methods | 57 (41.9%) | 106 (54.4%) | 0.034 |
| Professional correspondence | 95 (69.9%) | 126 (64.3%) | 0.348 |
| Time/project management | 104 (76.5%) | 134 (68.7%) | 0.156 |
| Effective meeting strategy | 57 (41.9%) | 93 (47.7%) | 0.354 |
| Scientific communication | 101 (74.3%) | 149 (76.4%) | 0.751 |
| Presenting results | 100 (74.6%) | 157 (81.3%) | 0.187 |
| Using strong statistical voice | 75 (55.1%) | 118 (60.5%) | 0.389 |
| Collaboration with analytic colleagues | 60 (44.1%) | 68 (34.7%) | 0.105 |

**Caption:** Denominators for percentages include only the participants who responded with an importance rating for the given skill. P-value represents the association between highest degree earned and rating the skill as “Absolutely essential” (yes/no) from a chi-squared test.

**Supplemental Table 3**: Heterogeneity in rating each skill as absolutely essential by job sector

| **Skill** | **Academia (n=238)** | **Industry (n=76)** | **Government (n=15)** | **Self-employed (n=14)** | **p-value** |
| --- | --- | --- | --- | --- | --- |
| Databases, data sources, and data collection tools | 118 (51.3%) | 48 (65.8%) | 11 (73.3%) | 6 (46.2%) | 0.043 |
| Developing clinical/ scientific domain knowledge | 84 (36.5%) | 29 (39.2%) | 6 (40.0%) | 5 (38.5%) | 0.784 |
| Regulatory requirements | 45 (19.6%) | 27 (36.5%) | 4 (26.7%) | 3 (23.1%) | 0.005 |
| Institutional structure | 35 (15.3%) | 8 (10.8%) | 0 (0.0%) | 1 (7.7%) | 0.443 |
| Statistical analysis plans | 167 (72.6%) | 53 (71.6%) | 14 (93.3%) | 6 (46.2%) | 0.987 |
| Reproducibility | 138 (60.0%) | 42 (56.8%) | 10 (66.7%) | 6 (46.2%) | 0.720 |
| Coding | 176 (76.9%) | 59 (79.7%) | 13 (86.7%) | 9 (69.2%) | 0.723 |
| Literature review | 42 (18.3%) | 13 (17.6%) | 3 (20.0%) | 3 (23.1%) | >0.999 |
| Learning new statistical methods | 119 (52.0%) | 32 (43.2%) | 7 (46.7%) | 5 (38.5%) | 0.242 |
| Professional correspondence | 156 (67.8%) | 49 (66.2%) | 10 (66.7%) | 6 (46.2%) | 0.909 |
| Time/project management | 166 (72.2%) | 55 (74.3%) | 11 (78.6%) | 6 (46.2%) | 0.833 |
| Effective meeting strategy | 104 (45.4%) | 30 (40.5%) | 8 (53.3%) | 8 (61.5%) | 0.549 |
| Scientific communication | 172 (74.8%) | 55 (74.3%) | 13 (92.9%) | 10 (76.9%) | >0.999 |
| Presenting results | 183 (80.6%) | 52 (71.2%) | 11 (78.6%) | 11 (84.6%) | 0.126 |
| Using strong statistical voice | 128 (55.9%) | 47 (63.5%) | 11 (73.3%) | 7 (53.8%) | 0.309 |
| Collaboration with analytic colleagues | 86 (37.4%) | 32 (43.2%) | 6 (40.0%) | 4 (30.8%) | 0.446 |

**Caption:** Denominators for percentages include only the participants who responded with an importance rating for the given skill. P-value represents the association between job sector (only academia vs. industry) and rating the skill as “Absolutely essential” (yes/no) from a chi-squared test. Government and self-employed job sectors are not included in the test of heterogeneity by job sector due to the small number of respondents.
